# Evaluation of the international standardized 24-h dietary recall methodology (GloboDiet) for potential application in research and surveillance within African settings

**DOI:** 10.1186/s12992-017-0260-6

**Published:** 2017-06-19

**Authors:** Elom Kouassivi Aglago, Edwige Landais, Geneviève Nicolas, Barrie Margetts, Catherine Leclercq, Pauline Allemand, Olaide Aderibigbe, Victoire Damienne Agueh, Paul Amuna, George Amponsah Annor, Jalila El Ati, Jennifer Coates, Brooke Colaiezzi, Ella Compaore, Hélène Delisle, Mieke Faber, Robert Fungo, Inocent Gouado, Asmaa El Hamdouchi, Waliou Amoussa Hounkpatin, Amoin Georgette Konan, Saloua Labzizi, James Ledo, Carol Mahachi, Segametsi Ditshebo Maruapula, Nonsikelelo Mathe, Muniirah Mbabazi, Mandy Wilja Mirembe, Carmelle Mizéhoun-Adissoda, Clement Diby Nzi, Pedro Terrence Pisa, Karima El Rhazi, Francis Zotor, Nadia Slimani

**Affiliations:** 1 Nutrition and Metabolism Section (NME), International Agency for Research on Cancer (IARC/WHO), 150 Cours Albert Thomas, 69372 Lyon, France; 2 UMR 204 ‘Nutripass’ IRD/UM/SupAgro, Montpellier, France; 3 Institute of Human Nutrition, University of Southampton, Southampton, UK; 4 Food and Agriculture Organization of the United Nations (FAO), Rome, Italy; 5 National Horticultural Research Institute, Ibadan, Nigeria; 6 Regional Institute of Public Health (IRSP), University of Abomey-Calavi, Cotonou, Benin; 7 Research Section, Primary Health Care Corporation, Doha, Qatar; 8 Department of Nutrition and Food Science, University of Ghana, Accra, Ghana; 9 INNTA, Tunis, Tunisia; 10 Gerald J. and Dorothy R. Friedman School of Nutrition Science and Policy, Tufts University, Boston, USA; 11 Department of Biochemistry-Microbiology, UFR-SVT, CRSBAN/University of Ouagadougou I Joseph Ki-Zerbo, Ouagadougou, Burkina Faso; 12 TRANSNUT, Centre Collaborateur OMS sur la transition nutritionnelle et le développement, Département de Nutrition, Faculté of Médecine, Université de Montréal, Montréal, Canada; 13 Non-Communicable Diseases Research Unit, South African Medical Research Council, Tygerberg, South Africa; 14 School of Food Technology, Nutrition & Bio-Engineering, Makerere University, Kampala, Uganda; 15 Faculty of Science, University of Douala, Douala, Cameroon; 16 Unité mixte de recherche en Nutrition et alimentation; URAC 39; RDC-Nutrition associé à l’AFRA/AIEA (CNESTEN-Université Ibn Tofail), Rabat, Morocco; 17 Department of Food Science and Nutrition, Faculty of Agricultural Science, University of Abomey-Calavi, Cotonou, Benin; 18 Université Félix Houphouët Boigny, Abidjan, Côte d’Ivoire; 19 Ministry of Health, Rabat, Morocco; 20 DietPlus Ghana, Accra, Ghana; 21 Department of Physiology, University of Zimbabwe, Harare, Zimbabwe; 22 Department of Family and Consumer Sciences, University of Botswana, Gaborone, Botswana; 23 Faculty of Health Disciplines, Athabasca University, School of Public Health, University of Alberta, Edmonton, Canada; 24 Division of Nutritional Sciences, School of Biosciences, University of Nottingham, Nottingham, UK; 25 Faculty of Epidemiology and Population Health, London School of Hygiene and Tropical Medicine, London, UK; 26 Faculty of Health Sciences, University of Abomey-Calavi, Cotonou, Benin; 27 Food and Agriculture Organization (FAO), Abidjan, Côte d’Ivoire; 28 Wits RHI, University of Witwatersrand, Johannesburg, South Africa; 29 Department of Epidemiology and Public Health, Faculty of Medicine of Fez, Fez, Morocco; 30 University of Health and Allied Sciences, Ho, Volta Region Ghana

**Keywords:** GloboDiet, Africa, 24-h dietary recall, Dietary assessment, Standardisation

## Abstract

**Background:**

Collection of reliable and comparable individual food consumption data is of primary importance to better understand, control and monitor malnutrition and its related comorbidities in low- and middle-income countries (LMICs), including in Africa. The lack of standardised dietary tools and their related research support infrastructure remains a major obstacle to implement concerted and region-specific research and action plans worldwide. Citing the magnitude and importance of this challenge, the International Agency for Research on Cancer (IARC/WHO) launched the “Global Nutrition Surveillance initiative” to pilot test the use of a standardized 24-h dietary recall research tool (GloboDiet), validated in Europe, in other regions. In this regard, the development of the GloboDiet-Africa can be optimised by better understanding of the local specific methodological needs, barriers and opportunities. The study aimed to evaluate the standardized 24-h dietary recall research tool (GloboDiet) as a possible common methodology for research and surveillance across Africa.

**Methods:**

A consultative panel of African and international experts in dietary assessment participated in six e-workshop sessions. They completed an in-depth e-questionnaire to evaluate the GloboDiet dietary methodology before and after participating in the e-workshop.

**Results:**

The 29 experts expressed their satisfaction on the potential of the software to address local specific needs when evaluating the main structure of the software, the stepwise approach for data collection and standardisation concept. Nevertheless, additional information to better describe local foods and recipes, as well as particular culinary patterns (e.g. mortar pounding), were proposed. Furthermore, food quantification in shared-plates and -bowls eating situations and interviewing of populations with low literacy skills, especially in rural settings, were acknowledged as requiring further specific considerations and appropriate solutions.

**Conclusions:**

An overall positive evaluation of the GloboDiet methodology by both African and international experts, supports the flexibility and potential applicability of this tool in diverse African settings and sets a positive platform for improved dietary monitoring and surveillance. Following this evaluation, prerequisite for future implementation and/or adaptation of GloboDiet in Africa, rigorous and robust capacity building as well as knowledge transfer will be required to roadmap a stepwise approach to implement this methodology across pilot African countries/regions.

**Electronic supplementary material:**

The online version of this article (doi:10.1186/s12992-017-0260-6) contains supplementary material, which is available to authorized users.

## Background

The global nutrition transition associated with an increasing prevalence of overweight and obesity is a real health and societal challenge affecting particularly low- and middle-income countries (LMICs) worldwide, including those in Africa [1–3]. This transition is characterized by rapid changes in diets shifting from a traditional and prudent high fibre, low-fat diet to a diet rich in saturated fat, animal source foods, salt, processed foods, added sugar, lower intake of fruits and vegetables, fibre and complex carbohydrates and increased consumption of meals eaten out-of-home, especially from fast foods chains [4–6]. In addition, physical activity has substantially decreased, especially in urban areas with increased sedentary behaviour in the secondary and tertiary economic sectors activities, as compared to primary sector activities which are more prevalent in rural areas [7]. This tangled situation has resulted in the steady increase in non-communicable diseases (NCDs) such as cardiovascular diseases, respiratory diseases, diabetes, and certain types of cancer in the LMICs [8, 9]. Concernedly, micronutrient deficiency is still tremendously prevalent within these populations, leading to a double burden of malnutrition difficult to be subdued by the fragile economy of the concerned countries [10, 11].

Accurate assessment and monitoring of malnutrition in LMICs, especially early detection of changes in the nutritional status of the populations, might require the collection of high-quality dietary data because individual food consumption is intimately related to the nutritional status. Dietary data can be collected for several purposes including setting of nutritional recommendations, exposure and safety assessment as well as overall nutritional surveillance.

We have previously illustrated from an inventory conducted in 18 African countries that various dietary assessment methodologies are used across the continent, predominantly for surveillance and research purposes [12]. Amongst these methodologies are the 24-h dietary recalls, food frequency questionnaires (FFQ), dietary records and dietary history. The 24-h dietary recall is an open-ended method intended to report detailed information about all foods and beverages consumed by a respondent, in the preceding 24 h or over the previous day [13, 14]. The interview can be paper-and-pencil-based, computer-assisted or rarely self-administrated. The method relies on short memories and allows the quantification of all the foods and beverages consumed over the period concerned.

Although only few of the used methodologies were reported to have been validated, their reported scientific outcomes have shaped the understanding of the food consumption landscape in Africa. However, in practice, there is a need to appraise the validity of the methodologies used and strengthen individual food consumption data collection and comparability throughout Africa. The optimal way to achieve this goal is to build on pre-existing experience and accelerate transfer of knowledge and capacity building among researchers and countries, considering the specific scientific, social, cultural and ethical African context. Diverse initiatives have already been launched on the continent to cover this gap. Although these initiatives do not strictly embed the same objectives, they act as fundamental components to cover the food consumption collection field in Africa. For more than six decades, dietary intakes activities in Africa have been pioneered by diverse institutions including the Food and Agriculture Organization of the United Nations (FAO), Harvard University, Wageningen University and the French National Research Institute for Sustainable Development (IRD) through the development of country- or region-specific food composition tables (http://www.fao.org/infoods/infoods/tables-and-databases/africa/en/), the training of local research teams, as well as the designing of studies on dietary assessment methods use in epidemiologic studies [15, 16]. The International Network of Food Data Systems (INFOODS, http://www.fao.org/infoods/infoods/en/) benefitted from these experiences, and through its regional data centre AFROFOODS published the West-African food composition table ^(^[17]^)^and developed relevant courses [15]. Besides this, the FAO/WHO Global Individual Food consumption data Tool (FAO/WHO GIFT, http://www.fao.org/nutrition/assessment/food-consumption-database/en/) aimed at increasing the harmonization and availability of existing data. The International Dietary Data Expansion (INDDEX, www.inddex.nutrition.tufts.edu) project led by Tufts University was designed to increase the availability, access, and use of household and individual food consumption data in low-income countries by developing open source and open access tools for dietary data collection, processing, analysis, and policy application.

The International Agency for Research on Cancer (IARC), the affiliated specialised institute of the WHO on cancer research, has developed a 24-h dietary recall software for the collection of standardised dietary data for epidemiological, surveillance and monitoring purposes [18]. A series of quality control measures are utilized from the preparation of the databases to data collection and management [19]. The software based on the GloboDiet (previously EPIC-Soft) methodology, has already been used in 19 European countries for both research and surveillance purposes. In addition, the GloboDiet methodology has successfully been adapted in Latin America (Brazil) and in the Republic of Korea. The specific adaptations needed for this first Asian country were recently published [20].

The implementation of GloboDiet in Africa will require adaptation based on prior in-depth understanding of the specific context of the African continent, with its diverse populations, languages, food sources and habits. Through a consultation panel consisting of both African and international experts with known and recognised experience in the field of dietary assessment, this paper reports on the evaluation findings of GloboDiet’s interview steps and other various aspects towards a feasible platform to be used for surveillance, monitoring and research purposes in diverse African settings.

## Methods

### The GloboDiet interview

The evaluation sessions covered all the sections of an interview conducted using the 24-h dietary recall GloboDiet application. The main sections of the dietary interview comprise (i) the general information on the interviewee, (ii) a quick list of the foods and recipes consumed the preceding day, (iii) the description using facets (questions) and descriptors (answers), (iv) the quantification, (v) probing questions, (vi) final controls, (vii) and information on the dietary supplements consumed [21]. The chronological description of the GloboDiet interview sections is presented in Table 1.

**Table 1:** Structure of an interview by GloboDiet

Section	Description	Features^a^
General information	Non-dietary information for the identification of the participant and information on the recalled day	Name, code, birth date, sex, anthropometric data, date of interview and wakeup time
		Special diets and special days^b^(e.g. gluten free, veganism, energy restricted) and (e.g. feast day, travel, illness, holidays)
Quick list	Open list to collect foods and beverages consumed at each occasion (cognitive approach, qualitative information)	Consumption occasions^b^ (e.g. before breakfast, breakfast, during the morning, lunch, dinner, after dinner, during the evening)
		Consumption time (hour with/without minutes)
		Places of consumption^b^: (e.g. home, work place, catering, fast food, bar, café, pub, street)
Description of foods and recipes	Search and detailed description of the foods and recipes consumed using facets and descriptors	e.g. Facets (and descriptors)^b^
		Food preparation and purchase (prepared at home, commercial, restaurant, vending machine, fast food)
		Cooking method (raw, fried, battered and fried, baked, sautéed, stewed, boiled, barbecued, steamed)
		Physical state (liquid, powdered, reconstitution from powdered)
		Fat content (whole, fat reduced, light)
Quantification of foods and recipes	Quantification of the described foods and recipes using photos, shapes, HHMs, SU, reported weight or volume, standard portions	Quantification methods with possibility to select a fraction or a multiple^b^
		Photos (e.g photos of a salad)
		Shape (e.g portion of a pie)
		HHMs (e.g. photos of graduated spoons, bowls, plates, glasses)
		SU (an apple, a can)
		Mass or volume method (in g, ml)
		Standard portions (e.g. melted cheese on a dish)
		Unkwown^c^ (fats, sauces and sweeteners)
Probing questions	Checklist during the quick list step and after quantification of items to recall the interviewer of easily forgettable foods in link with other consumed foods	e.g. Food (and probing)^b^
		Tea (sugar)
		Bread (topping)
	Recall on non-filled quick list items and daily energy and macronutrients aberrant values	e.g. Daily calories intake (too low or too high)
	Warning maximum exceed	e.g. Volume of milk > 600 ml
	Note entering^d^	
		e.g. Volume of 1000 ml of milk have been confirmed by the subject
Dietary supplements	Recall on dietary supplements taken: search, description and information on quantification	Vitamins, minerals or oligoelements capsules

*HHM* household measure, *SU* standard unit

^a^non exhaustive list

^b^ad hoc, to be adapted for each local situation/project

^c^Standard percentages of fats, sauces, sweeteners

^d^Automatic or manual

### Preparatory phase of the e-workshop

**Fig. 1:**
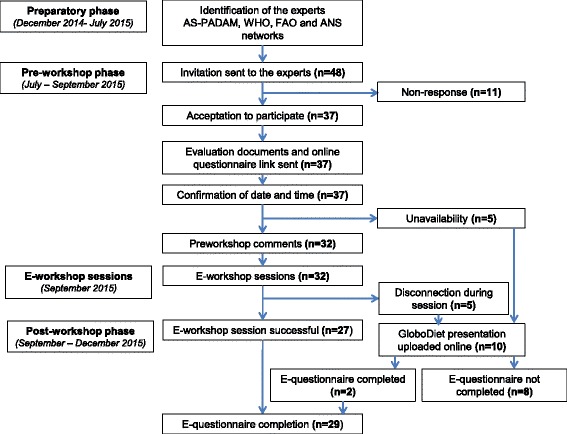
Flow chart for the participation in the evaluation. A total of 48 identified experts from various networks were invited, amongst whom 32 were available to participate in a session of the e-workshop. During the e-workshop sessions, the experts disconnected [5] were re-invited to watch online uploaded video and comments afterwards. A total of 29 experts completed the final questionnaire

The experts who participated in the Africa’s Study on Physical activity and Dietary Assessment Methods (AS-PADAM) coordinated by IARC [12], served as preliminary list. This preliminary list was constituted as a team of food and nutrition experts who were selected based on accomplished knowledge in their areas of excellence. International institutions (WHO, FAO) and the African Nutrition Society (ANS) proposed experts from their networks using a snowball sampling method to reach a total of 48 African and international experts from universities and research institutes. The invitation of the experts did not foresee any statistical representativeness by country or region, but was rather constituted as a consultative panel of African and international experts with expertise in dietary assessment, and eventually surveillance or implementation of diet-related programs at country- or regional level. Nonetheless, it was empirically decided to include at least one African expert from each UN sub-region as dietary habits and research issues might differ between these regions. Figure 1 summarizes the diagram flow of how the experts identified were invited for the evaluation of GloboDiet.

### Pre-workshop phase

The identified experts were invited through email and three dates with sessions scheduled in the morning and the afternoon of each date were proposed for the evaluation. Core documents including a (i) scientific paper presenting the GloboDiet software [18], (ii) a presentation on the methodology and (iii) 30 min video simulating interaction between an interviewer and an interviewee were provided to each participating expert to get acquainted with the methodology. An additional folder consisting of poster presentations and published papers on the structure, evaluation, standardization, validation, quality insurance and other GloboDiet components was assembled and uploaded online for participants to study in preparation of the workshop [18–27].

In parallel, an e-questionnaire was developed using Wepi™, a simplified online questionnaire authoring and publishing application for health professionals (EpiConcept, www.wepi.org). The e-questionnaire consisted of nine parts that covered all the aspects of the GloboDiet interview. The first three parts focused on geographical, institutional, and socio-demographic information on the experts as well as pre-workshop questions and comments that the participants might have on reading the documentation that was sent to them. The fourth to sixth parts were dedicated to the evaluation of the different GloboDiet interview sections. The last three parts evaluated the overall controls, probing questions, dietary supplements and a general evaluation of the software. Four types of questions were included in the questionnaire: dichotomous (e.g: Is there any missing food description that would be relevant in the African context? - Yes/No), Likert scale types (e.g. How do you evaluate the pictures of foods for the face-to-face interviews? It is convenient to use? Strongly agree/Agree/Neither agree nor disagree/Disagree/Strongly disagree), contingency questions (e.g. If you think the pictures of foods are not applicable for use in Africa, could you specify why?), and open questions (e.g. Which adaptations of the pictures of household measures could you think of to make it more suitable for African settings?). An individual link as well as an ID code to keep anonymity was sent to each expert. The experts were encouraged to fill the first three parts of the e-questionnaire to facilitate the preparation of the e-workshop session they would participate in.

### E-workshop sessions

Six identical e-workshop sessions of 3 hours each were conducted: four in English and two in French to comply with adequate language proficiency for all the experts. The sessions were conducted through Citrix GoToMeeting (Citrix, CA, USA), an online conference platform. Four to six experts participated in each session. Sessions proceeded as follows: brief introduction of each participant, presentation of the GloboDiet initiative and the GloboDiet software, followed by an open discussion. During the presentation of the interview procedure, participants were invited to ask questions, add comments and suggestions through the inset “Chat” windows of the GoToMeeting. Questions and comments were gathered and addressed during the open discussion.

#### Post-workshop phase

After the workshop, the experts were invited to complete all the parts of the e-questionnaire, if not already done or/and add further comments. Some of the participants who initially agreed to take part in the e-workshop sessions and who were finally unable to participate were invited to watch an online uploaded video of a session, and ultimately completed the online questionnaire afterwards. Four experts from Tufts University and FAO (two from each) completed one questionnaire per institution.

### Data analysis

Quantitative data collected through the e-questionnaires were analysed using statistical distribution. Qualitative information from the e-questionnaires and sessions reports were summarized into general ideas, and the core information was assorted to other responses provided by the experts for the same topics. The results of the analysis were drafted in a paper to the writing group to distil redundant and irrelevant information. The Likert scale plot was performed using R 3.0.1 (R Foundation for Statistical Computing, Vienna, Austria).

## Results

### Participants

General characteristics of the experts that were invited to take part in the evaluation are reported in Table 2. Of the 48 African and international experts invited, 29 experts participated in the sessions and completed the e-questionnaire. Eighteen experts participated in the four sessions in English while 11 participated in the two sessions in French. A total of 19 experts were unable to participate in the e-workshop sessions: 11 did not reply to the two invitation emails, five were unable to participate due to their personal unavailability to attend one of the proposed sessions, five were unable to connect during the e-workshop sessions, or disconnected during the presentations, amongst whom two further became participants after watching the presentation uploaded online and filling the questionnaires.

**Table 2:** General characteristics of the panel of experts

	Number
Gender	
Male	9
Female	20
All	29
Age group	
20–29	1
30–39	13
40–49	4
50–59	8
>60	3
Education	
Master	7
PhD or MD	22
Affiliation	
Academic institution	20
Global institution	4
Research centres	3
Ministry of Health	1
Not known^a^	1
Type of dietary methodology expertise	
24-h dietary recall	29
Food frequency questionnaire	25
Dietary record	18
Dietary history	9

^a^Missing data

African participant experts were from 13 countries: three from Eastern Africa (Uganda), one from Middle Africa (Cameroon), four from Northern Africa (Morocco, Tunisia), five from Southern Africa (Botswana, South Africa, Zimbabwe), and nine from Western Africa (Benin, Burkina Faso, Côte d’Ivoire, Ghana, Nigeria). Seven experts were from international institutions (WHO, FAO) and universities outside Africa (Tufts University, USA; University of Alberta, Canada; University of Southampton, UK).

Table 3 summarizes the opinions and recommendations regarding all the different sections of the GloboDiet 24-h recall interview, and the possibility of addressing or adapting them into the software in the future. In order to keep the methodology standardized at the international level, the African GloboDiet version should align with common rules already endorsed in all the countries that adopted the GloboDiet methodology but new specifications or adaptations could be developed if necessary.

**Table 3:** Experts evaluation of GloboDiet sections and response address

Section	Overall evaluation^a^	More specific suggestions	Response approach in GloboDiet
General information	Adequate, useful, applicable, comprehensive, easily understandable, complete, excellent, simple, concise and relevant	Additional: dwelling place, marital status, number of children, education, physical activity, breastfeeding status, employment status	Actually handled by additional questionnaire(s) (non-dietary data to be merged with dietary data)
		Adaptation: delete participant name, measure weight and height instead of self-reporting	Local interview training issue
		Ask age instead of birth date, non-consideration of sickness and travel days as special days, ask for diet-related diseases directly	These requests can be addressed in GloboDiet
Quick list	Easy, comprehensive, good, useful features, clear, appropriate, detail-focused, intuitive, interesting	Adaptation: ask for daily activities of the interviewee to capture food consumption occasions	Adaptation of food consumption occasions list and local training issue
Description of foods and recipes	Relevant aspects covered, detailed, comprehensive, clear	-	Adaptation of the foods and recipes lists
	Necessity to contextualize	Additional: sun drying, smoking, mortar and peddle pounding, stone grounding, sifting, gifts, home production	Study how to link to existing GloboDiet facets/descriptors
			Possibility to add new descriptors and facets.
		Adaptation: possibility to delete packaging, all the facets related to canned fruits and vegetables	Adaptation of the facets and descriptors databases to local situations
Quantification of foods and recipes	Satisfactory (see Fig. 2)	Adaptation: Clear distinction between urban and rural areas	Adaptation of the quantification methods to local settings
		Have pictures of local foods without fork and knife, local HHMs	Create local picture books with local HHMs, foods/recipes,
			Use local standard units
		SU (be sure about the cultivars), real foods, handful and soft food scoping	Quantification method to be linked to volume
		Food models, salted replicas,	Weight method
		Onsite weighing	New quantification approach to be defined.
		Shared plate	Study/validation of method “shared plate”.
Probing questions	Important, relevant	Additional: wild foods and fruits picking	Actually handled at the quick list step
			Training issue
Quality controls	Needed, important	-	Well-grounded in GloboDiet
Dietary supplements	Relevant, adapted, necessary, good, helpful, appropriate	Definition of dietary supplements is not clear in Africa	There is need to clearly define dietary supplements before inclusion in Globodiet databases (as food, dietary supplements, or both)
		Additional: “tonic” and “energy-booster” plant by-products	

*HHM* household measure, *SU* standard unit

^a^As commented by the experts in the online questionnaire

### General information

Comments on the general information focused on its easiness with most experts reporting its relative completeness. A few comments pointed out the need to include more information on the socio-economic and physiological status of the interviewee. Self-reporting of birth and anthropometric measures was questioned, especially in the context of low literacy. To note, the general information available in GloboDiet is flexible and can allow addition and removal of specific data. The general information can be retrievable in the personal sheet of participants in a research study. Therefore, it was explained that the general information section does not intend to be exhaustive but rather to give information on the interviewee. Nevertheless, minimal data (age and sex) are needed for final controls that are based on energy requirement.

### Quick list

As individual food consumption occasions must comply with the activities undertaken in a day, some experts proposed to capture the daily activities of the interviewee and then construct foods consumption around it. This approach may reveal at the beginning of the interview, times and places of consumption. To the experts panel, the chronological approach for the quick list in GloboDiet reinforces the intuitiveness to capture foods consumed by day, and is a validated approach to screen a whole day and track all possible foods consumed. In the case this approach is not adapted to spot all food consumption occasions in specific settings, this can be addressed by training of the interviewers to exhaustively collect foods consumed, place of consumption, time and occasion.

### Description of foods and recipes

There was consensus among the experts that the facet/descriptor approach (i.e. systematic predefined food−/recipe-questions asked and their related answers) was an appropriate and detailed way to standardize food item descriptions. In the African context, there was consensus that the contrast between urban and rural areas requires slightly different facets and descriptors of foods and recipes. There was an agreement to retain some facets already present in GloboDiet because they describe individual food consumption patterns in urban areas. The situation is quite different in rural areas where new descriptors such as sun drying, smoking, mortar pounding and grinding would be relevant. Descriptors “dried”, “smoked” are already available in GloboDiet in the facet preservation method. Likewise, the descriptor “pureed/mashed” is present in the facet physical state. Adding and/or removing facets and descriptors is feasible in GloboDiet. Concretely, an agreement on necessary facets and descriptors will have to be found for the African setting for incorporation into the software.

### Quantification of foods and recipes

**Fig. 2:**
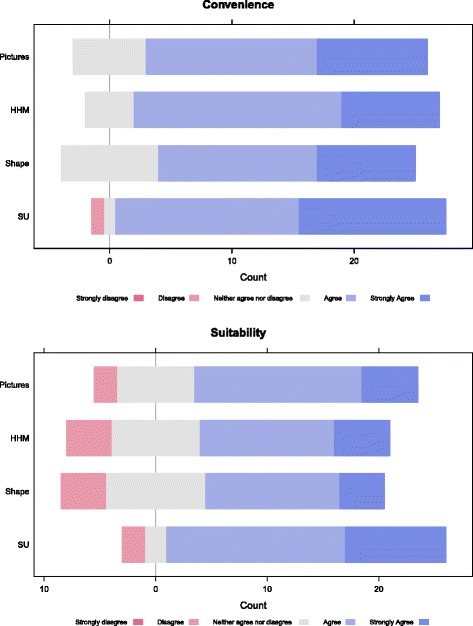
Appreciation of quantification methods used in GloboDiet. The comments of the experts on the quantification methods available in GloboDiet were satisfactory. Standards units were evaluated as the most convenient method followed by picture books and household measures. Likewise, the three methods were considered as the most applicable in the African context. Overall, shape quantification was considered as the least applicable in Africa

The panel of experts acknowledged overall that the quantification methods used in GloboDiet, notably the household measures, the pictures, the standard units, and the food shapes, are suitable for African settings (Fig. 2). In this regard, some adaptations were highlighted which are part of the general customisation procedure to be followed for the preparation of any GloboDiet version. Firstly, selected local foods and recipes need to be photographied and composed as pictures books which would be tested and validated as portion size estimation aids in rural contexts where pictoral literacy is likely to be low. In addition, these pictures could be used to identify the items consumed in low literacy settings. Secondly, specific African household measures (HHMs) should be inventoried and included in the database for quantification. Thirdly, shape/volume quantification from a pictures book was identified as the least convenient method and would require specific attention for more adaptation. Lastly, standard units (SU) should consider the different cultivars of fruits for example or be matched with photos showing sizes/shapes for both identification and quantification. It is indeed a usual prerequisite to include local SU in GloboDiet databases. In addition, the experts have proposed other methods of quantification which have been tested and used in African rural areas: real foods, food models or salted replicas as spatial models might facilitate the quantification step in rural areas.

### Probing and overall controls

There was unanimity among the experts regarding the importance of the probing section in the 24-h recall methodology, as embeded in GloboDiet. Reasons provided were the need to check the interviewee’s responses as well as the necessity to provide pop ups to bring to the interviewer’s attention for some accompanying foods and toppings. As already in GloboDiet, experts acknowledged that a probing should be neutral. The African version of GloboDiet should consider probing about easily forgettable items such as wild foods and fruits picking when completing check list or during the inventory of food consumption occasions. Overall controls are flexible, and energy and macronutrients cut-offs can be adapted according to a population of interest or for a study.

### Dietary supplements

The general impression of the experts on the dietary supplements section was positive. Approximately half (*n* = 14) of the experts estimated that the proposed separation of the dietary supplements from foods and recipes is important in order to repertoriate, describe and quantify them properly. The rest of the experts (*n* = 15) suggested, based on the potential confusion between supplements and some medications in Africa, to conduct in advance an extensive search to identify, describe and classify dietary supplements before further decisions for use and inclusion in dietary assessement in general, and in GloboDiet in particular. Therefore, there is first a need of clarification for what should be considered as dietary supplements in Africa. Thereafter, there will also be a need to cover specific African supplements used as “tonic” or “energy-booster” which are prepared from plant by-products. These suggestions were retained as specific requirements to dapt the GloboDiet and its databases in the African context.

## Discussion

The GloboDiet methodology designed for nutritional research and surveillance based on the 24-h dietary recall is a tool that has, initially, been used to collect dietary consumption data in the EPIC study. The cumulative experience that went into the development, testing, validation and implementation of this international software enables the establishment of strong science-based evidence on its reliability, detailed information, robustness and flexibility that made it possible to be adapted amongst other countries and contexts [23, 27]. Likewise, the main feature of the GloboDiet 24-h dietary recall software is its design that ensures a high level of standardisation of data collection, management and analyses within and between countries. Therefore, the consultation of the panel of African and other experts was an efficient way to share information on the methodology and receive feedback on the potential of the existing version and additional adaptations needed for Africa.

Within the GloboDiet software, the general information on the interviewee (e.g. age, sex, height and weight) is important for final controls on the reported total energy and macronutrient intakes vs. recommendations, as well as for individual identification information (e.g. interviewee’s identification code). It should be recommended that in the context of low-literacy, anthropometric measures be entered from a participant sheet to avoid self-declared information, which is a plausible source of errors [28, 29]. The “special days” within the general information might also concern local market days in rural Africa, because foods available in households and consumed on that day are more diverse, and do not reflect routine consumption [30]. In addition, these foods available and then consumed that day fluctuate with the purchasing power of the households.

To fully describe a consumed item, a combination of two food description pathways is used in GloboDiet. The implicit pathway uses the name of the foods as self-standing (e.g. *injera* uniquely could refer to the Ethiopian sourdough bread made of *teff*), whereas the explicit description requires facets and descriptors (e.g. *injera* may require at least the facets: food production/purchase location, salt content). The use of facets and descriptors privileged in GloboDiet is also used in food description worldwide by organisations such as the European Food Safety Authority [31]. Through the consultation of the experts, it was confirmed that most of the facets/descriptors available in GloboDiet European versions are also suitable for the description of foods and recipes consumed in urban areas in Africa. However, as in any preparation of a GloboDiet version, typical African foods will need to be included in GloboDiet databases and combined implicit and explicit descriptions will be applied to each food and recipe during data collection. Additional descriptive pieces of information that may be deemed necessary to collect are sources of drinking water [32, 33] and water treatment methods [34, 35] because drinking water is still a vector of microbial infections and chemical hazards in Africa. Other examples of description consideration details that might be interesting to consider are application of treatments to decrease cyanogenic agents in cassava [36], or the covering of street-vended foods. It is also important to consider food fortification status in the description of the foods available in Africa since several foods and staples are now subjects to mandatory or voluntary food fortification programs in Africa [37]. Food fortification should be included in African databases if interviewees are likely to recall the food items and/or if fortified foods could be recalled during the interview and dealt a *priori*.

The major challenge in food quantification that was raised unanimously by the experts is the individual consumption quantification from a shared plate, considered as a notable issue in studies using the 24-h recall [38, 39]. To address this issue, some researchers reported the distribution of HHMs beforehand by field enumerators, and asked the participants to serve individual portions [40, 41]. However, Kigutha [42] hypothesized that this might affect eating habits of the subjects, and therefore introduce biases in individual intake estimation. Asobayire [43] quantified individual intake by weighing participants before and after eating and adjusting for beverages volume consumed in separate bowls. This approach, besides weight measurement constraints and errors, is only valid to estimate the consumption of a single food and recipe, without accurate quantification of diverse item consumed. In the Gambia, an algorithm has been applied to approximate individual portion consumed from a shared bowl considering body weight, sex and age [44]. The formula used in this algorithm has not been tested in other settings, suggesting that, if an algorithm is the chosen approach to solve the shared plate issue, there is a need to find a systematic and reliable algorithm to estimate individual intake from shared-plate.

Besides the consumption from a shared plate, food quantification methods usually applied in GloboDiet have been acknowledged by the panel of experts as valid for quantification of African foods and recipes. Pictures have been extensively used for quantification in Africa [45] and validation studies have been carried out [46, 47]. Picture atlas in GloboDiet is a series of a minimum of four high-quality unpixellated coloured photos at incremental food sizes. Adaptation of the existing GloboDiet photos to the African context will require updating of the foods and recipes to be photographed and the deletion of the cutlery not in use in African settings. Food grids developed and validated by GAIN for the Food Assessment Coverage Toolkit (FACT) could be utilized as a primary tool [48] and used a complement to validated picture of foods for the quantification of individual intake. Likewise, previous studies suggest that HHMs including cups, mugs, spoons and plates are extensively used for quantification in Africa [49–53]. Adaptation of the HHMs booklet will include photos of local HHMs, but also having these onsite during the interviews to ease quantification. However, in contrast to foods pictures and HHMs, shape/volume quantification appears to be laborious because misrepresentation of the actual size of the foods may occur in relation to the visual perception of the subjects. Shapes are predominantly used in GloboDiet to quantify breads and wedge-shaped portions and have not been extensively used in Africa, which imply a thorough analysis of applicability and adaptability. Overall, there is a necessity to validate the combination of methods of quantification proposed by Willett [13] comprising household measures, photographs, geometric shapes, standard units, and other innovative methods likely to be developed in the very context of LMICs. In addition to individual portion size estimation, memory lapses have been reported as substantial sources of errors in food intake assessment in Africa [54]. Therefore probing and various controls are important to capture all the foods consumed, their description and quantification in order to prevent misreporting. GloboDiet contains a series of probing and control steps from database preparation, data collection, calculation and data cleaning in order to decrease these errors [19]. Apart from usual probing such as sugar for tea and topping for breads, African-specific probing can be included according to specific behaviours in some African regions. For example, condiments such as the stock cubes which are ubiquitously found in African countries can be candidate for probing. Another important issue in Africa would be the need to extensively train field enumerators for the collection of individual food consumption data, considering the local environment.

### Strengths and limitations of the study

To our knowledge, this study is the first to conduct an e-workshop gathering a panel of African and other international experts in dietary assessment through Internet. Despite the low Internet penetration in Africa, this study overcame the shortage through beforehand steady preparation and special assistance that guided the experts throughout the whole process of e-workshop with direct phone calls when needed. Furthermore, the possibility to conduct sessions in French permitted to include a larger number of French-speaking experts who might have been impeded with probable English proficiency issues. Although, we failed to recruit Spanish- and Portuguese-speaking experts in addition, we assume that this has a little impact on the consultation reported in this study because their regions are covered.

One of the limitations of this study was the difficulty to align the agenda of all the experts invited to the workshop. Despite the fact that it would have been preferable to have more experts participating in the e-workshop sessions, the e-workshop successfully gathered experts from all the African five United Nations sub-regions, suggesting that the necessary adaptations required for GloboDiet are compiled; In-depths national or local requests could be addressed during implementation.

## Conclusion

The contributions of the experts were constructive and will serve as the foundation for the possible future implementation of the GloboDiet methodology in Africa. It can be summarized that successful and standardised assessment of individual food consumption across Africa is possible but would need the inclusion and combination of description and quantification methods available in GloboDiet, as well as the specific propositions drawn from this consultation. Furthermore, particular attention should be given to the shared plate consumption, to improve quantification of individual intake. Also, a clear definition of dietary supplements will be necessary to handle the African specific situation.

The possible implementation of the GloboDiet methodology in the African context will be an opportunity for public health, nutrition, food safety and cancer researchers to collect and analyse high-standard quality food consumption data. Preliminary work to assess the needs and gaps to adapt the GloboDiet methodology is a prerequisite before any roadmap for implementation. Africa is a heterogeneous continent constituted of more than 50 countries with various dietary cultures and the most efficient approach will be to develop several versions of GloboDiet-Africa either by country or by region, with few pilot countries as starting point. Further steps will therefore include, but not exclusively: collection of foods and recipes from African surveys, detailing of appropriate facets and descriptors, exploration of suitable quantification pathways and their validation in pilot studies.

Africa needs to collect regular data on nutrition and its determinants, as a starting point of ingrained nutrition surveillance programs capable to support the continent in the process of fulfilling the Sustainable Development Goals (SDGs). Overall, concerted actions will be needed and collaboration between and with African networks will be the key to successful implementation.

## Additional files

Additional file 1: Answers of the e-questionnaire by the experts panel. (XLSX 46 kb)

Additional file 2: Evaluation questionnaire in English. (PDF 505 kb)

Additional file 3: Evaluation questionnaire in French. (PDF 145 kb)

## Abbreviations

ANS: African nutrition society
AS-PADAM: Africa’s study on physical activity and dietary assessment methods
FAO: Food and agriculture organization of the United States
FFQ: Food frequency questionnaire
HHM: Household measure
IARC: International agency for research on cancer
INDDEX: International dietary data expansion
INFOODS: International network of food data systems
LMIC: Low- and middle-income countries
NCD: Non-communicable diseases
SDG: Sustainable development goals
SU: Standard unit
WHO: World health organization
