# Supplementary material for: Evaluation of the international standardized 24-h dietary recall methodology (GloboDiet) for potential application in research and surveillance within African settings
Source: Global Health. 2017 Jun 19;13:35. doi: 10.1186/s12992-017-0260-6 (PMC5477249; doi:10.1186/s12992-017-0260-6)
Supplement: Supplementary file 2 — Evaluation questionnaire in English. (PDF 505 kb) [file 12992_2017_260_MOESM2_ESM.pdf]

EVALUATION OF THE GLOBODIET BY AFRICAN EXPERTS

**Identification**	
First name:<br>Family name:<br>*The above information will not be computerized*	Participant ID<br>*do not fill this box*<br>|__|__|__|
1. Institution:	|__|
2. Country:<br>3. WHO region:<br>☐1 EMRO ☐2 AFRO-West<br>☐3 AFRO-central ☐4 AFRO-East-South	|__| |__|

**Socio demographic information**	
4. Gender<br>☐1 Male ☐2 Female	|__|
5. Age group<br>☐1 20-29 ☐2 30-39 ☐3 40-49<br>☐4 50-59 ☐5 ≥60	|__|
6. Education:<br>☐1 A level ☐2 BSc or equivalent ☐3 MSc or equivalent<br>☐4 PhD	|__|
7. Occupation :	|__|
8. What kind of dietary methods are you familiar with (in the sense of previously used)?<br>☐1 24 h dietary recall ☐2 FFQ ☐3 dietary records<br>☐4 dietary history	|__|
9. For the dietary methods you are familiar with, what was the main support?<br>☐1 paper ☐2 computer ☐3 Internet<br>☐4 tablet computer	|__|

General information
10. What do you think about the general information section?<br><br><br><br>11. Is there any additional information that you think would be relevant to add?

Quick list
12. What do you think about the quick list section?<br><br><br><br>13. Are there any changes you could think of to improve this section?<br><br><br><br>14. Are there any needed changes to adapt the quick list to African settings you could think of?

Description and Quantification	
15. What do you think about the food description?	|__| |__| |__| |__|
16. Is the food description adapted to African context?<br>☐0 No ☐1 Yes<br><br>**If no**, could you specify why?	|__| |__| |__|
17. Is there any missing food description that would be relevant for African context?<br>☐0 No ☐1 Yes<br><br>**If yes**, could you specify?	|__| |__| |__|
18. Are there any facet descriptors that are not relevant to African context?<br>☐0 No ☐1 Yes<br><br>**If no**, could you specify why?	|__| |__| |__|
19. Are there any missing facet descriptors that would be relevant for the African context?<br>☐0 No ☐1 Yes<br><br>**If yes**, could you specify?	|__| |__| |__|

20. How do you evaluate the p**ictures of foods for the face-to-face interviews**? It is:

	Strongly agree	Agree	Neither agree nor disagree	Disagree	Strongly disagree
convenient to use	☐1	☐2	☐3	☐4	☐5
applicable for use in African settings	☐1	☐2	☐3	☐4	☐5

21. If you think the **pictures of foods** are not applicable for use in Africa, could you specify why?	|__| |__|

|__|

22. Which adaptations of the **pictures of foods** could you think of to make it more suitable for African settings?

|__|

|__|

|__|

23. How do you evaluate the **pictures of household measures for the face-to-face interviews**? It is:

	Strongly agree	Agree	Neither agree nor disagree	Disagree	Strongly disagree
convenient to use	☐1	☐2	☐3	☐4	☐5
applicable for use in African settings	☐1	☐2	☐3	☐4	☐5

24. If you think the **pictures of household measures** are not applicable for use in Africa, could you specify why?

|__|

|__|

|__|

25. Which adaptations of the **pictures of household measures** could you think of to make it more suitable for African settings?

|__|

|__|

|__|

26. How do you evaluate the **shape quantification method for the face-to-face interviews**? It is:

	Strongly agree	Agree	Neither agree nor disagree	Disagree	Strongly disagree
convenient to use	☐1	☐2	☐3	☐4	☐5
applicable for use in African settings	☐1	☐2	☐3	☐4	☐5

27. If you think the **shape quantification method** is not applicable for use in Africa, could you please specify why?

|__|

|__|

|__|

28. How do you evaluate the **use of standard units** (e.g one banana, one can, one bag of potato chips,…) **as a quantification method for the face-to-face interviews**? It is:

	Strongly agree	Agree	Neither agree nor disagree	Disagree	Strongly disagree
convenient to use	☐1	☐2	☐3	☐4	☐5
applicable for use in African settings	☐1	☐2	☐3	☐4	☐5

29. If you think the **standard units quantification method** is not applicable for use in Africa, could you please specify why?

|__|

|__|

	|__|
30. Are there any other methods of quantification used or relevant in African settings that are not taken into account by GloboDiet software?<br>☐$_0$ No ☐$_1$ Yes<br><br>If yes, please specify:	|__
__
__	

**Overall controls**	
31. What do you think about the overall controls section?<br><br><br><br>32. What do you think about the probing questions?	|__
__
__
__
__
__	

Dietary supplements	
33. What do you think about the dietary supplement section?	|__| |__| |__|

Overall evaluation of the software

1. How do you evaluate realization of the **face-to-face computerized 24-h dietary recalls**? Do you think it is:

	Strongly agree	Agree	Neither agree nor disagree	Disagree	Strongly disagree
feasible	☐1	☐2	☐3	☐4	☐5
convenient	☐1	☐2	☐3	☐4	☐5
efficient	☐1	☐2	☐3	☐4	☐5
acceptable time load	☐1	☐2	☐3	☐4	☐5
not adapted to African settings	☐1	☐2	☐3	☐4	☐5

2. If you think the face-to-face interview is not adapted to the African settings, could you please specify why:

|__|

|__|

|__|

3. How do you evaluate the use of the **data entry application** (using food diary) for young children and elders? Do you think it is:

	Strongly agree	Agree	Neither agree nor disagree	Disagree	Strongly disagree
feasible	☐1	☐2	☐3	☐4	☐5
convenient	☐1	☐2	☐3	☐4	☐5
efficient	☐1	☐2	☐3	☐4	☐5
acceptable time load	☐1	☐2	☐3	☐4	☐5
not adapted to African settings	☐1	☐2	☐3	☐4	☐5

4. If you think the face-to-face interview is not adapted to the African settings, could you please specify why:

|__|

|__|

|__|

5. How do you evaluate the **GloboDiet 24-h dietary recall software for performing 24-h dietary recalls**? It is:

	Strongly agree	Agree	Neither agree nor disagree	Disagree	Strongly disagree
user-friendly	☐1	☐2	☐3	☐4	☐5
understandable	☐1	☐2	☐3	☐4	☐5
complete	☐1	☐2	☐3	☐4	☐5
complex	☐1	☐2	☐3	☐4	☐5
not adapted to African settings	☐1	☐2	☐3	☐4	☐5

6. If you think the software is not adapted to African settings, could you please specify why:

|__|

|__|

					|__|
7. How do you evaluate the use of a computer-based interview performed by an interviewer?					
	Strongly agree	Agree	Neither agree nor disagree	Disagree	Strongly disagree
feasible	☐1	☐2	☐3	☐4	☐5
convenient	☐1	☐2	☐3	☐4	☐5
adapted to African settings	☐1	☐2	☐3	☐4	☐5
8. If you think the computer-based interview is not adapted to African settings, could you please precise why:					|__| |__| |__|
