# Supplementary material for: Evaluation of the international standardized 24-h dietary recall methodology (GloboDiet) for potential application in research and surveillance within African settings
Source: Global Health. 2017 Jun 19;13:35. doi: 10.1186/s12992-017-0260-6 (PMC5477249; doi:10.1186/s12992-017-0260-6)
Supplement: Supplementary file 3 — Evaluation questionnaire in French. (PDF 145 kb) [file 12992_2017_260_MOESM3_ESM.pdf]

EVALUATION DE GLOBODIET PAR DES EXPERTS AFRICAINS ET INTERNATIONAUX

**Identification**	
Nom :	|__|__|__|
Prénoms :	
1. Institution:	|__|
2. Pays:	|__|
3. Région OMS:<br>☐1 EMRO ☐2 AFRO-Ouest<br>☐3 AFRO-centrale ☐4 AFRO-Est-Sud	|__|

**Informations sociodémographiques**	
4. Sexe<br>☐1 Homme ☐2 Femme	|__|
5. Classe d'âge (années)<br>☐1 20-29 ☐2 30-39 ☐3 40-49<br>☐4 50-59 ☐5 ≥60	|__|
6. Education:<br>☐1 Niveau A ☐2 Licence ou équivalent ☐3 Master ou équivalent ☐4 Doctorat	|__|
7. Occupation :	|__|
8. Avec quels types de méthodes de consommation alimentaire êtes-vous familier? (que vous avez déjà utilisé ?)<br>☐1 Rappel de 24 h ☐2 Fréquencier ☐3 Enregistrements alimentaires<br>☐4 Historique de consommation	|__|
9. Pour la méthode de consommation alimentaire que vous utilisez, quel était le support principal ?<br>☐1 papier ☐2 ordinateur ☐3 Internet<br>☐4 tablette	|__|

General Information (Information Générale)
10. Que pensez-vous de la section –General information?<br><br><br><br>11. Y-a-il une information supplémentaire pertinente que vous voudriez suggérer?

Quick list (Liste rapide)
12. Que pensez-vous de la section – quick list?<br><br><br><br>13. Y-a-il de possibles modifications susceptibles d’améliorer cette section ?<br><br><br><br>14. Existe-t-il de possibles modifications pour adapter la –quick list- au cadre Africain?

Description et Quantification	
15. Que pensez-vous de la description des aliments?	|__| |__| |__| |__|
16. La description des aliments est-elle adaptée au contexte Africain ?<br>☐0 Non ☐1 Oui<br><br>**Si non**, Pouvez-vous préciser pourquoi?	|__| |__| |__|
17. Y-at-il un critère de description des aliments non énuméré et pourrait être pertinent dans le contexte africain ?<br>☐0 Non ☐1 Oui<br><br>**Si Oui**, Pouvez-vous préciser pourquoi?	|__| |__| |__|
18. Y-at-il des facettes de description qui ne sont pas applicables dans le contexte Africain ?<br>☐0 Non ☐1 Oui<br><br>**Si oui**, Pouvez-vous préciser lesquels?	|__| |__| |__|
19. Y-a-il des facettes non énumérées, mais qui pourraient être applicables dans le contexte Africain ?<br>☐0 Non ☐1 Oui<br><br>**Si oui**, Pouvez-vous préciser pourquoi?	|__| |__| |__|

20. Comment évaluez-vous les photos des aliments pour l'interview face-à-face ? C'est :

	Très vrai	Vrai	Ni vrai Ni faux	Faux	Très faux
Pratique à utiliser	☐1	☐2	☐3	☐4	☐5
Applicable dans le contexte africain	☐1	☐2	☐3	☐4	☐5

21. Si vous pensez que l'utilisation des photos des aliments n'est pas applicable dans le contexte Africain, pouvez-vous préciser pourquoi?	|__| |__| |__|

22. Quelle suggestions pour améliorer les photos des aliments, pensez-vous pour mieux l’adapter au contexte africain?	|__| |__| |__|

23. Comment évaluez-vous les photos des **mesures ménagères** pour l’interview face-à-face? C’est :

	Très vrai	Vrai	Ni vrai Ni faux	Faux	Très faux
Pratique à utiliser	☐$_1$	☐$_2$	☐$_3$	☐$_4$	☐$_5$
Applicable dans le contexte africain	☐$_1$	☐$_2$	☐$_3$	☐$_4$	☐$_5$

24. Si vous pensez que les photos des **mesures ménagères** ne sont pas applicables dans le contexte africain, pouvez-vous préciser pourquoi?	|__| |__| |__|
25. A quelles adaptations des photos des mesures ménagères pensez-vous pour mieux l’adapter au contexte Africain?	|__| |__| |__|

26. Comment évaluez-vous la quantification par **les formes** pour l’interview face-à-face ? C’est

	Très vrai	Vrai	Ni vrai Ni faux	Faux	Très faux
Pratique à utiliser	☐$_1$	☐$_2$	☐$_3$	☐$_4$	☐$_5$
Applicable dans le contexte africain	☐$_1$	☐$_2$	☐$_3$	☐$_4$	☐$_5$

27. Si vous pensez que la quantification par **les formes** n’est pas applicable au contexte Africain, pouvez-vous préciser pourquoi?	|__| |__| |__|

28. Comment évaluez-vous l’utilisation des **unités standards** (Ex: une banana, une cannette, un sachet de chips, …) comme méthode de quantification pour l’interview face-à-face?

	Très vrai	Vrai	Ni vrai Ni faux	Faux	Très faux
Pratique à utiliser	☐$_1$	☐$_2$	☐$_3$	☐$_4$	☐$_5$
Applicable dans le contexte africain	☐$_1$	☐$_2$	☐$_3$	☐$_4$	☐$_5$

29. Si vous pensez que les **unités standards** comme méthode de quantification ne sont pas applicables au contexte africain, pouvez-vous préciser pourquoi?	|__| |__| |__|
30. Y-at-il une autre méthode de quantification utilisable ou pertinent dans le contexte Africain qui n’est pas prise en compte par le logiciel GloboDiet?<br>☐$_0$ Non ☐$_1$ Oui<br><br>**Si oui**, Pouvez-vous préciser?	|__| |__| |__|

Overall controls (Contrôle généraux)	
31. Que pensez-vous de la section -overall controls-?<br><br>32. Que pensez-vous des questions de vérification (probing questions)?	|__| |__| |__| |__| |__| |__|

Dietary supplements (Compléments alimentaires)	
33. Que pensez-vous de la section compléments alimentaires ?	|__
__
__	

Evaluation générale du logiciel					
1. Comment évaluez-vous la réalisation d'un entretien rappel 24h en utilisant un logiciel sur ordinateur ? Pensez-vous que c'est :					
	Très vrai	Vrai	Ni vrai Ni faux	Faux	Très faux
Faisable	☐1	☐2	☐3	☐4	☐5
Pratique	☐1	☐2	☐3	☐4	☐5
Efficace	☐1	☐2	☐3	☐4	☐5
Acceptable (durée)	☐1	☐2	☐3	☐4	☐5
Pas adapté au cadre Africain	☐1	☐2	☐3	☐4	☐5
2. Si vous pensez que l'entretien face-à-face n'est pas adapté au contexte Africain, pouvez-vous préciser pourquoi?					|__| |__| |__|
3. Comment évaluez-vous l'utilisation d'un logiciel pour l'enregistrement des données des consommations alimentaires pour les jeunes enfants et les personnes âgées ? Pensez-vous que c'est :					
	Très vrai	Vrai	Ni vrai Ni faux	Faux	Très faux
Faisable	☐1	☐2	☐3	☐4	☐5
Pratique	☐1	☐2	☐3	☐4	☐5
Efficace	☐1	☐2	☐3	☐4	☐5
Acceptable (durée)	☐1	☐2	☐3	☐4	☐5
Pas adapté au cadre Africain	☐1	☐2	☐3	☐4	☐5
4. Si vous pensez que l'interview face-à-face n'est pas adapté au contexte Africain, pouvez-vous préciser pourquoi?					|__| |__| |__|
5. Comment évaluez-vous le logiciel GloboDiet pour la réalisation d'un rappel de 24h? C'est :					
	Très vrai	Vrai	Ni vrai Ni faux	Faux	Très faux
Facile à utiliser	☐1	☐2	☐3	☐4	☐5
Compréhensible	☐1	☐2	☐3	☐4	☐5
Complet	☐1	☐2	☐3	☐4	☐5
Complexe	☐1	☐2	☐3	☐4	☐5
Pas adapté au cadre Africain	☐1	☐2	☐3	☐4	☐5
6. Si vous pensez que le logiciel n'est pas adapté au contexte Africain, pouvez-vous préciser pourquoi ?					|__| |__| |__|
7. Comment évaluez l'utilisation d'un logiciel installé sur ordinateur, avec entretien face-à-face pour évaluer la consommation alimentaire ?					
	Très vrai	Vrai	Ni vrai Ni faux	Faux	Très faux
Faisable	☐1	☐2	☐3	☐4	☐5

Pratique	☐1	☐2	☐3	☐4	☐5
Adapté au contexte Africain	☐1	☐2	☐3	☐4	☐5
8. Si vous pensez que l'entretien face-à-face n'est pas adapté au contexte Africain, pouvez-vous préciser pourquoi?					|__| |__| |__|
